# An experimental phantom study of the effect of gadolinium-based MR contrast agents on PET attenuation coefficients and PET quantification in PET-MR imaging: application to cardiac studies

**DOI:** 10.1186/s40658-017-0173-8

**Published:** 2017-01-13

**Authors:** Jim O’ Doherty, Paul Schleyer

**Affiliations:** 1 PET Imaging Centre, Division of Imaging Sciences and Biomedical Engineering, King’s College London, King’s Health Partners, St. Thomas’ Hospital, 1st Floor, Lambeth Wing, Westminster Bridge Road, London, SE1 7EH UK; 2 Siemens Healthcare Limited, Frimley, Camberley, UK

**Keywords:** PET-MR, Attenuation correction, Cardiac PET

## Abstract

**Background:**

Simultaneous cardiac perfusion studies are an increasing trend in PET-MR imaging. During dynamic PET imaging, the introduction of gadolinium-based MR contrast agents (GBCA) at high concentrations during a dual injection of GBCA and PET radiotracer may cause increased attenuation effects of the PET signal, and thus errors in quantification of PET images. We thus aimed to calculate the change in linear attenuation coefficient (LAC) of a mixture of PET radiotracer and increasing concentrations of GBCA in solution and furthermore, to investigate if this change in LAC produced a measurable effect on the image-based PET activity concentration when attenuation corrected by three different AC strategies.

**Findings:**

We performed simultaneous PET-MR imaging of a phantom in a static scenario using a fixed activity of 40 MBq [18 F]-NaF, water, and an increasing GBCA concentration from 0 to 66 mM (based on an assumed maximum possible concentration of GBCA in the left ventricle in a clinical study). This simulated a range of clinical concentrations of GBCA. We investigated two methods to calculate the LAC of the solution mixture at 511 keV: (1) a mathematical mixture rule and (2) CT imaging of each concentration step and subsequent conversion to LAC at 511 keV. This comparison showed that the ranges of LAC produced by both methods are equivalent with an increase in LAC of the mixed solution of approximately 2% over the range of 0–66 mM.

We then employed three different attenuation correction methods to the PET data: (1) each PET scan at a specific millimolar concentration of GBCA corrected by its corresponding CT scan, (2) each PET scan corrected by a CT scan with no GBCA present (i.e., at 0 mM GBCA), and (3) a manually generated attenuation map, whereby all CT voxels in the phantom at 0 mM were replaced by LAC = 0.1 cm^−1^. All attenuation correction methods (1–3) were accurate to the true measured activity concentration within 5%, and there were no trends in image-based activity concentrations upon increasing the GBCA concentration of the solution.

**Conclusion:**

The presence of high GBCA concentration (representing a worst-case scenario in dynamic cardiac studies) in solution with PET radiotracer produces a minimal effect on attenuation-corrected PET quantification.

## Introduction

Gadolinium-based contrast agents (GBCA) represent the most common types of magnetic resonance contrast agents, used primarily as a T1 contrast agent. GBCA consist of transitional (i.e., heavy) metal Gd ions bound by chelating agents to form a stable complex of relatively low toxicity [1]. Many GBCA have different molecular structures yet similar pharmacokinetic properties, and therefore, few differences can be discerned in clinical practice [2]. Paramagnetic ions such as Gd^3+^ in GBCA dissolved in an aqueous solution act as microscopic magnets in the local environment causing water protons to “feel” a large magnetic moment and thus a local change in the average relaxation time. They are most commonly employed due to a predominant shortening of T1 relaxation time, which results in an increased signal intensity on a T1-weighted image (known as positive enhancement).

The use of simultaneous PET-MR (positron emission tomography-magnetic resonance) in cardiology opens up the potential for the simultaneous injection of a PET perfusion tracer (such as [15O]-H2O, [^13^N]-NH_3_, or [^82^Rb]-Cl) with GBCA for parallel myocardial perfusion quantification using both methodologies. Also, as cardiac MR imaging is prone to scanner-dependent calibration curves and saturation effects [3, 4], this quantification methodology could also allow direct comparison between calculated PET and MR perfusion variables and quantification techniques [5].

Previous investigations of the effect of GBCA in clinical PET-MR imaging have shown that MR-based attenuation maps acquired via a two-point VIBE-based DIXON sequence (whereby an automated segmentation algorithm provides four different tissue classes: fat, soft tissue, lung, and air) may be affected only by orally administered iron-oxide-based contrast agent and that neither intra-venous injections nor orally administered GBCA significantly affect the attenuation of the PET emission data [6]. This group’s work looked at clinically relevant concentrations of GBCA for static whole-body imaging, determining a worst-case scenario for the concentration in the blood. However, not yet investigated are the technical considerations for a dynamic simultaneous PET-MR acquisition, such as those required for calculation of an image-derived input function and myocardial uptake curves in PET cardiology studies.

In this work, we aimed to assess the effects of high concentrations of GBCA firstly on the change in linear attenuation coefficient (LAC—the fraction of photons absorbed per unit thickness of the material) of a solution of increasing GBCA concentration and PET radiotracer and secondly on PET image-based activity concentration. We employed CT imaging and a mathematical model to provide measurements of the LAC at 511 keV, as well as investigated the effects of any change in LAC on the quantification of PET image-based activity concentration using three different attenuation correction methods.

## Material and methods

### Solution preparation

In order to simulate a “worst-case scenario” of the maximum possible GBCA concentration in the left ventricle of the heart during clinical imaging, an assumption was made that an entire bolus of GBCA can be present in the left ventricle. Thus, we assumed a maximum bolus volume of 20 ml being diluted in an average end diastolic left ventricle volume (EDV) of 150 ml (142 ± 21 ml is a reported EDV range [7]). Assuming 20 ml of 0.5 mmol/ml solution GBCA in the left ventricle, the molar concentration of GBCA (from Table 1) can reach a potential maximum of approximately 70 mM. After ejection of the GBCA from the heart, the concentration in the left ventricle then quickly reduces (over approximately 30–50 s) as it distributes into a larger blood volume. Thus, our static experiments aimed to cover the minimum to potential maximum range of GBCA concentrations in the left ventricle during the times that both MR and PET arterial input functions are measured on resulting reconstructed images.

**Table 1:** The composition of common MR contrast agents in terms of the amount of Gadolinium present in the solution from the summary of product characteristics datasheets

Parent solution	Active component	Molecular weight of active component (g/mol)	Mass of active component in 1 ml of parent solution (mg)	Mass of Gd in 1 ml of parent (mg)
DOTAREM® 0.5 mmol/ml	Gadoteric acid	558.64	279.32	78.625
Gadovist ®1 mmol/ml	Gadobuterol	604.71	604.71	157.25
Magnevist ® 0.5 mmol	Gadopentetic acid	545.56	469.01	78.625
MultiHance® 0.5 mmol	Gadobenic acid	667.72	529.00	78.625

Added chelator may be present around the Gd complex. The molar mass of gadolinium is 157.25 g/mol

A thin plastic bottle (max volume = 160 ml, *d* = 5 cm, *h* = 8.5 cm) was filled with 120 ml of distilled water together with 40 MBq of [^18^F]-NaF in 0.2 ml (as measured in a standard dose calibrator with ±5% accuracy) in order to provide measurements of PET activity concentration.

We then added DOTAREM 0.5 mmol/l [8]—a GBCA utilized throughout our hospital—in incremental 3-mM steps until a 30-mM solution was reached (ten concentration steps). After reaching 30 mM, 4-mM steps (ten steps in total) were added to make a solution with final concentration of 66 mM. At each concentration step, the solution was scanned on a CT scanner followed by a PET-MRI scanner.

### Scanning

CT images were acquired only for calculation of the LAC of the solution on a GE Discovery 710 PET-CT scanner (140 kV, 20 mA, 0.5-s rotation, 40-mm collimation). No PET scanning was performed on the PET-CT scanner. PET-MR scans were performed on a simultaneous whole-body PET-MR scanner (Siemens Biograph mMR, Siemens Healthcare, Erlangen, Germany) located next door to the PET-CT scanner. Each PET-MR scan lasted 3 min, and all PET data was decay corrected to a common time point.

By default, during PET scanning, an MR-based attenuation correction (MRAC) sequence was performed with each PET-MR scan at each GBCA concentration step. This was generated using the standard dual-point VIBE T1-weighted Dixon sequence provided by the manufacturer on the scanner.

### Mixture rule for calculation of LAC

**Fig. 1:**
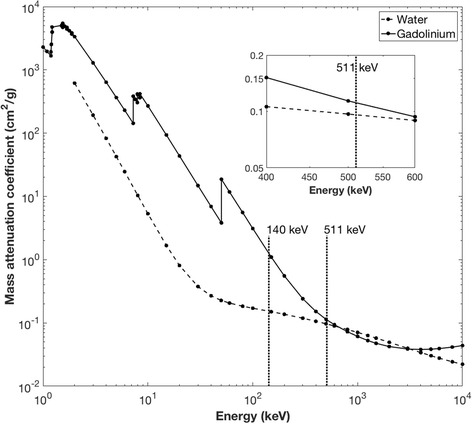
Mass attenuation spectra of water and gadolinium, with a line drawn at 511 keV showing the similar mass attenuation coefficients. *Inset* shows a close-up of the values at 511 keV. At 500 keV (the last measured point), this difference in *μ*_M_ is 14.95%. Data has been replotted from tabulated data originally published by Hubbell [9]

In order to understand how the introduction of GBCA can affect the image-based PET activity concentration during simultaneous PET-MR, it is important to understand the attenuation properties of the different components at 511 keV. Data for the mass attenuation coefficient (MAC—characterizes how easily the material is penetrated by gamma radiation) of Gd and water are shown in Fig. 1 [9]. At 150 kV (close to the CT energy of 140 kV), markedly different MACs of 1.1 and 0.1505 cm^2^/g for Gd and water, respectively can be observed. However, at 500 kV (close to PET gamma energy of 511 keV), these MACs are more similar, 0.1139 and 0.0969 cm^2^/g for Gd and water, respectively. The measured LAC of other tissues of the body at 511 keV are also similar at this energy [9] (skeletal muscle = 0.1010 cm^−1^ [10], adipose tissue = 0.09 cm^−1^ [11], and whole blood = 0.0905 cm^−1^ [11]).

The MAC of a homogenously mixed solution can be approximated by Hubbell’s weighted average mixture rule for homogenous solutions with photon energies >10 keV [9]:

1 $$ {\mu}_{\mathrm{M}\left(\mathrm{soln}\right)}={\displaystyle {\sum}_i{\mu_{\mathrm{M}}}_{(i)}}{w}_{(i)} $$

where *μ*_M(soln)_ represents the MAC of the total solution and *μ*_M*(i)*_ and *w*_*(i)*_ represent the MAC (cm^2^/g) and fractional weight of the *i* ^th^ components of the mixture. Given that the solution of GBCA can be approximated to a mixture of gadolinium (Gd) and water (wa), this can be written as:

2 $$ {\mu}_{\mathrm{M}\left(\mathrm{soln}\right)}={\mu}_{\mathrm{M}(wa)}{w}_{(wa)}+{\mu}_{\mathrm{M}\left(\mathrm{G}\mathrm{d}\right)}{w}_{\left(\mathrm{G}\mathrm{d}\right)} $$

Thus assuming that the measured values of MAC at 500 keV are representative of those at 511 keV, in order to determine the MAC, and hence, the LAC (LAC = MAC * solution density), the total mass of solution and fractional weights of water and gadolinium are required. Given that *μ*_M(wa)_ and *μ*_M(Gd)_ at 511 keV are 0.9687 and 0.1139 cm^2^/g, respectively (from Fig. 1), the final mixture will have *μ*_M(soln)_ confined to *μ*_M(Gd)_ > *μ*_M(soln)_ *> μ*_M(wa)_ *.*

### Image reconstruction and analysis

#### Investigation of LAC

All CT images were reconstructed on the PET-CT scanner using a filtered back-projection (FBP) algorithm as standard on the scanner software. Transformation from Hounsfield units (HU) to LAC at 511 keV was performed offline using a bi-linear calibration curve (140 kVp) as implemented on the PET-CT scanner. LAC values applied to the images by the MRAC segmentation procedure (each voxel in the image represents LAC × 10,000) were obtained from the MR attenuation map by viewing the images on the scanner software and noting down the common LAC value applied to each voxel of the solution in the phantom.

#### PET quantification

In order to quantify any effect, a change in GBCA concentration (and hence a change in LAC) of the solution may have on final PET image data, attenuation correction of the PET data is required. All PET data were reconstructed on the PET-MR scanner using standard clinical reconstruction parameters (OSEM, 3 iterations, 21 subsets, 344 image matrix). PET data was not reconstructed using the default MRAC algorithm provided on the scanner of each GBCA step, instead PET emission sinograms were attenuation corrected with three different methods:

1. AC1—Each PET image corrected by its corresponding CT-derived attenuation map. Each CT-derived attenuation map was registered to the MR-derived attenuation map using a rigid registration through Niftyreg software [12] and subsequently uploaded to the PET-MR scanner for attenuation correction of PET data.
2. AC2—A CT-derived attenuation map with LAC values resulting from a CT scan of the phantom at 0 mM (i.e., no GBCA present). The dataset was registered and uploaded to the scanner as described for method AC1.
3. AC3—A manually generated attenuation map whereby all CT voxels in the phantom were manually set to 0.1 cm^−1^.

Method AC1 provides a standard method for attenuation correction, given that the LAC calculated from the bi-linear scaling of CT data from each GBCA step is being used to correct its corresponding PET scan. Method AC2 is employed as is common in a clinical scenario, where a single MR attenuation map acquired before the injection of PET radiotracer and GBCA is used to attenuation correct all dynamic PET frames. Method AC3 represents a scenario of using a single “soft tissue” LAC value as would be assigned by the MRAC segmentation algorithm on clinical scanning.

All PET and CT image analyses were performed in OsiriX [Pixmeo SARL, Geneva, Switzerland]. A rectangular volume of interest (VOI) corresponding to a central portion of the solution was drawn on the phantom (volume = 75 cm^3^) at each concentration step. The average HU, image-based PET activity concentration (kBq/ml), and VOI standard deviations were obtained from the relevant slices (28 CT slices, 50 PET slices). Resulting PET data were decay corrected to a common time point and were also corrected for the increasing volume of the solution in order to visualize differences from the true activity concentration and from the LAC of the solution at 0-mM concentration.

## Results

### Investigation of LAC

**Fig. 2:**
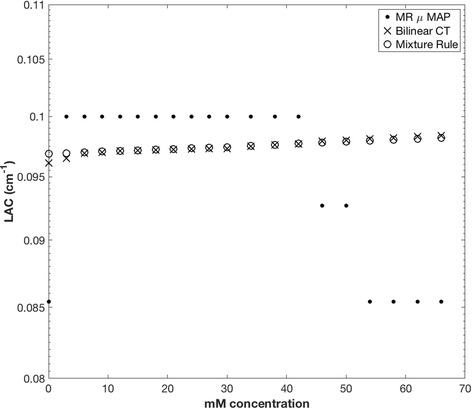
The LAC as determined by bi-linear CT calculation and the theoretical mixture model. MRAC-derived LAC values are shown for comparison only and were not used to correct PET data. CT and mixture model are closely correlated, showing an increase of approximately 2% up to 66 mM. The MRAC segmentation routine fails to determine accurate LAC at higher mM concentrations due to T1-shortening effects caused by the presence of high concentrations of GBCA

Figure 2 shows a comparison of LAC with increasing GBCA concentration for the mixture rule (Eq.2) and resulting LAC from CT scanning (bi-linear conversion from HU to LAC at 511 keV). LACs as generated by the MRAC segmentation are also shown for comparison only. LACs of the solution generated from CT imaging show a maximum increase of approximately 2% over the range of 0 and 66 mM, which correlates well with the increase predicted from the mixture model as described above.

### Effect on PET quantification

**Fig. 3:**
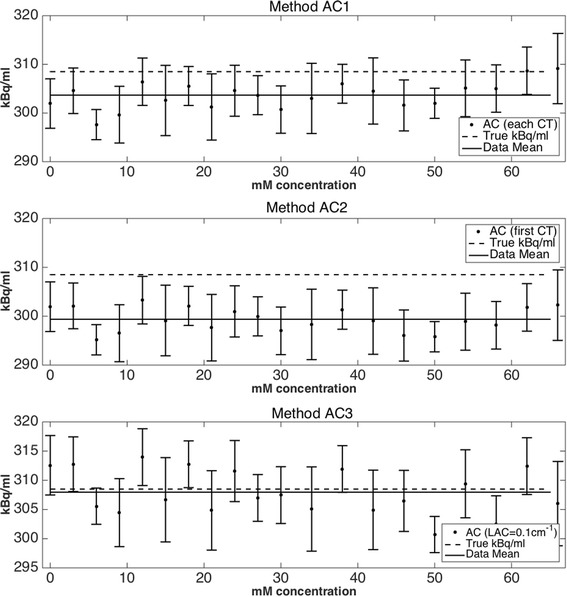
Comparison of decay-corrected and volume-corrected image-based PET activity concentrations. PET data acquired on the PET-MR system were attenuation corrected by three different methods: (*top*) AC1 using the CT scan from each increasing step in GBCA, (*middle*) AC2 by using the first CT scan with no GBCA present, and (*bottom*) AC3 from a manually generated attenuation map where all voxels have LAC = 0.1 cm^−1^. Error bars represent one standard deviation of the VOI used to calculate the mean of PET data. No trends in mean image-based activity concentration can be observed while increasing GBCA concentration for any of the attenuation correction methods

Figure 3 shows the effect of the different attenuation correction strategies (AC1, AC2, and AC3) on the quantification of PET data. The true activity concentration in the phantom was calculated at each time point from the knowledge of the original activity placed in the phantom, compensated for decay, and also the increasing volume at each concentration step. The image-based activity concentration is comparable across all three attenuation correction methods, and no trends are visible with increasing GBCA. Error bars in the activity concentration represent the mean kBq/ml ± one standard deviation of the mean, in order to indicate the level of noise present in the resulting images. It should be noted that in dynamic imaging a higher level of noise is likely to be obtained due to short frame times (potentially as short as 5–10 s depending on the imaging protocol), and low noise here indicates good count statistics only.

## Discussion

Our primary goal in these experiments was to evaluate the change in LAC of a mixture of PET radiotracer and increasing concentrations of GBCA, and also to investigate if this change produces a measurable effect on the image-based PET activity concentration.

### Investigation of LAC

As proposed by Fig. 1, the effect of increasing concentrations of GBCA on quantification of image-based PET activity concentration should be limited to a very small range between the MAC of water and gadolinium at a photon energy of 511 keV.

As detailed in Fig. 2, LAC comparisons via bi-linear CT closely match the LAC values resulting from the mixture rule calculations (Eq.2) with an increase of approximately 2% in LAC over the increasing GBCA range of 0 to 66 mM. This details that, in general, the mixture model can be utilized to predict the LAC of a solution of water and GBCA for phantom studies. Erroneous values of LAC derived from the MRAC segmentation procedure are shown in Fig. 2 for comparison to the data derived from the mixture model and from CT imaging only. Studies have shown in vivo the T1- and T2-shortening effects due to the use of GBCA in contrast enhancement studies, with a range from 30 to 68% shortening of T1 post administration of 0.1 mmol/kg body weight [13]. The effect of GBCA on clinically derived MR attenuation maps has recently been demonstrated [14], showing an overestimation of image-based activity concentration due to an assignment of part of the lung tissue to the soft tissue by the MRAC due to the presence of GBCA. This produced a measurable effect due to the large difference in LAC between the lung and soft tissue. In a simultaneous PET-MRI clinical cardiac acquisition, the AC procedure would be free from the influences of GBCA if the MRAC scan were performed before the administration of GBCA. However, if additional MRAC are performed after GBCA administration, effects of GBCA on the segmentation algorithm have to be taken into account.

### Effects on PET quantification

Figure 3 details the accuracy of the correction strategies (AC1, AC2, and AC3) to the true activity concentration of each solution. We did not employ attenuation correction via the default MRAC procedure due to the inaccuracies of the MRAC in defining LAC of the solution as detailed above. All three AC methodologies were within 5% of the ground truth activity concentration, although AC3 gave the most accurate mean image-based activity concentration over all solutions to the true value. Values consistently lower than the true activity concentration were a maximum of 2.5% and are likely to originate from the calibration factor between the dose calibrator and PET scanner, although this value is well within the locally set tolerance of 5%. A mean difference of 1.5% was observed between PET data corrected by methods AC1 and AC2. Method AC3 represents the closest to a clinical approximation, as this is the determined LAC of the “soft tissue” class from MRAC segmentation, and would be applied to the heart and its contents in a clinical cardiac PET-MR acquisition. Although an LAC of 0.1 cm^−1^ was manually applied to the phantom data to simulate the value applied to the heart by the MRAC segmentation, method AC3 is valid only for this phantom setup as the effects of segmentation and LAC determination of structures external to the phantom (such as non-cardiac tissues in a clinical scenario) were not investigated in this work. It is important to note also that the solution in our study was water mixed with tracer and GBCA, rather than blood (MAC_BLOOD_ = 0.0959 cm^2^/g at 511 keV), which may produce a different effect on LAC determination from the automatic segmentation routine.

Our assumption of all of the GBCA pooling in the left ventricle together with the radiotracer is likely to be an overestimation of the true scenario. In practical circumstances, the GBCA in cardiac MR studies is injected at a rate of 3 ml/s. With a standard heart rate of 60 bpm, GBCA would be cleared rapidly from the ventricle, indicating that the true GBCA concentration during a dynamic acquisition is likely to be a lot lower that 66 mM. However, we have addressed a broad concentration range of GBCA up to this maximum point.

Static imaging, in order to investigate the effects of GBCA on image-based activity concentration, was performed in order to control all parameters except GBCA concentration rather than the use of a dynamic phantom whereby concentrations of both GBCA and PET radiotracer are both changing rapidly. The use of a dynamic cardiac perfusion phantom for investigations into quantification of MR cardiac perfusion studies [15] would allow the investigation of the attenuation effects on dynamically acquired PET and MR input functions. Use of such high concentration of GBCA (66 mM) may lead to effects of signal saturation (itself potentially corrected for by adjustment of the magnetization flip angle in gradient echo sequences [16]) in the derivation of an MR input function, the effects of which could also be investigated with a phantom. Furthermore, the use of an anthropomorphic torso phantom with cardiac insert could provide a more realistic comparison to a clinical scenario (i.e., such as scattering of gamma ray photons). This would have required regular access to the cardiac chamber of the phantom which was impractical with the amount of steps of increasing concentration used in this study. Also, the study was concerned mainly with a carefully controlled study of the quantitative accuracy of PET when mixed in solution with GBCA, and thus, a true patient representation was not required.

In order to avoid the potential confounding effects of dead time on the PET scanner when all of the radiotracer is placed in the field of view of the scanner, we utilized a PET activity of [18 F]-NaF of 40 MBq. This represents an activity far lower than that usually received by patients at our center undergoing [13 N]-NH3 cardiac imaging. The effect of dead time has been quantified on previous cardiac studies on PET-CT systems, for example, the effect on myocardial perfusion quantification [17], and also the limit of dead time losses by weight-based activity administration protocols [18]. Dead time effects have yet to be investigated in cardiac PET-MR imaging. As this work investigated the effect of GBCA on image-based measurements of PET activity concentration, the total activity in the phantom is not an important factor, as any GBCA effect would have the same contribution regardless of the total activity. We also aimed to reduce the radiation dose to the operator as much as possible due to multiple handling, filling, and transport steps performed.

## Conclusion

Our work employed a static simulation of a bolus of gadolinium-based contrast agent (GBCA) in solution with water and PET radiotracer in a simulated left ventricle. Our results have shown that when considering high concentrations of up to 66 mM of GBCA, the linear attenuation coefficient (LAC) of the mixed solution increases by approximately 2% over the 0–66 mM range. The quantitative accuracy of the resulting reconstructed PET images when attenuation corrected by CT data, and also a manually applied attenuation map is minimally affected by the presence of the GBCA.
